# Quantum Rate Dynamics for Coherent Electron Transport at Material/Electrolyte Interfaces

**DOI:** 10.1021/acsami.5c25018

**Published:** 2026-02-13

**Authors:** Paulo Roberto Bueno

**Affiliations:** Department of Physics and Mathematics, Institute of Chemistry, 153997 São Paulo State University, Araraquara 14800-060, São Paulo, Brazil

**Keywords:** Quantum Rate Theory, Electron Transport, Electron Transfer, Electrochemical Capacitance, Quantum Coherence, Material/Electrolyte Interfaces, Marcus Theory, Nanoscale Electronics

## Abstract

Nanoscale electronics and electrochemistry are both based on the fundamental principles of electron motion at the material/electrolyte interfaces. Despite this common ground, these fields use distinct conceptual frameworks: physicists favor coherent electron transport, while chemists rely on kinetic electron transfer. In this work, we present the fundamental quantum-mechanical principles that unify these approaches, linking quantum transport to the electron-transfer rate constant in an electrolyte environment. We show that—even at room temperature—electron motion between quantum states, which appears as a slow kinetic rate, is in fact driven by underlying coherent quantum dynamics, modulated by the electrolyte’s damping. This coherent transport determines the kinetics of redox switches, controls biological processes such as Geobacter respiration, enables the development of *in situ* spectroscopic techniques, and accounts for the charge dynamics observed in reduced graphene oxide supercapacitance. As a result, these approaches provide a way to measure the electronic structure of quantum dots and graphene at energies below the radio frequency range. In light of these findings, we discuss the limitations of the traditional reorganization energy (λ_0_), which has been used to quantify the low-frequency rate of reaction dynamics in electrochemistry, and propose its replacement with measurable quantum circuit parameters intrinsic to the material’s electronic structure.

## Graphical abstract

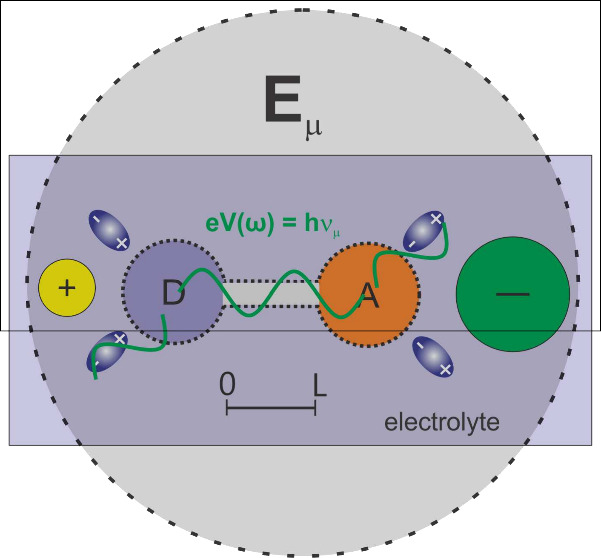

## 1 Introduction

The phenomenon of electricity has fascinated humanity for centuries. The fundamental relationship among charge transfer, electrical energy, and chemical processes lies beneath this fascination, particularly in biological chemistry, where electrons and ions play key roles. Various forms of electronics and biology depend on electron transfer reactions at interfaces and ion transport through solutions.

Salted solutions (electrolytes) provide an environment where the electron’s charge is shielded as it moves between quantum states. Recent advances show that, beyond ion-induced damping of electrodynamics at finite temperature, this phenomenon is quantum coherent.^1,2^ This ultimately clarifies and supports our understanding of how electric flow works quantum mechanically in chemistry and biology.^3^

This perspective reveals that it is this coherence that enables the unification of the different approaches chemists and physicists take to the phenomenon of electron motion. This unification can boost development in the field of nanoscale science and technology, which majorly depends on quantum mechanical principles encompassing electron motion.^4–11^ Quantum chemistry uses semiclassical approximations that serve quite well in most cases. In these cases, the photon field (light) is treated as a separate entity from the molecules,^12^ and light–matter interactions are not deeply integrated. For instance, quantum chemistry does incorporate quantum electrodynamics to study how molecules interact with electromagnetic fields in nanoscale cavities.^13–15^ However, comprehension of the principles behind the phenomena is not only still incipient in the field of molecular chemistry, particularly in the part that involves electron motion (i.e., electrochemistry^16^) but also in the field of nanoscale electronics^17^ performed in an electrolytic (‘wet’) environment. Nowadays, the fields of electrochemistry (chemistry) and nanoscale electronics (physics) deal with electron motion (or the exchange of electronic information) between quantum states in two different guises: electron transfer (chemists) and electron transport (physicists) viewpoints.

### 1.1 The Physicist’s Viewpoint: Electron Transport

The physicists consider an urgent task that affects the future of ‘dry’ nanoscale electronics. In the absence of electrolyte, this is referred to as nanoscale solid state physics or mesoscopic physics.^18^ This challenge is outlined in the roadmap for the semiconductor industry, which drives the development of computer processors, such as central processing units (CPUs) or coprocessors such as graphics processing units (GPUs). Currently, gate widths for CMOS (complementary metal-oxide-semiconductor) transistors are below 8 nm. Shortly, they are predicted to decrease by less than 2 nm. This is close to the limit where quantum effects, which are not yet completely understood, are dominant, and classical circuit laws fail.

For instance, devising an electronic device using molecules has been the goal in some areas of nanotechnology,^19–21^ and molecular electronics^21,22^ has been proposed as an alternative to silicon post-CMOS devices.^20,23^ The natural question in such a scenario is: Will molecular-based quantum devices replace traditional CMOS technology? The answer depends on researchers’ ability to understand quantum physics and control and fabricate devices at such a diminutive scale.

The cornerstone of modern electronics still relies on the ‘dry’ silicon technology, but elements of quantum conductance have been introduced in what is referred to as FinFET technology,^24^ which represents a leap in the design of the transistors, within an innovative three-dimensional architecture that permits a tangible link to the fundamental principles of quantum conductance *G*, as defined by Rolf Landauer’s seminal formula^25,26^

$$G=G_{0}\overset{N}{\underset{n}{\sum }}T_{n}$$ 1

where *G*_0_ = *g*_s_ *e* ^2^/*h* ≈ 77.5 μS is a universal constant referred to as the conductance quantum, where *e* is the elementary charge of the electron, *g*_s_ is the spin degeneracy, *h* is the Planck constant, and *T*_*n*_ is the electron’s transmission probability through a single quantum channel *n*. The Landauer formalism describes **coherent** transport, where the electron’s phase coherence is maintained across the channel. In this context, the term “Fin” refers to the thin vertical slice of silicon that rises from the substrate to form the channel of the transistor. The thickness of these fins, which function as quantum wires, is nowadays typically quite small—ranging from 5 to 10 nm—which confines electrons and leads to quantum mechanical effects. These effects are central to the device’s operation and pave the way for future innovations in this area.

### 1.2 The Chemist’s Viewpoint: Electron Transfer

Nanoscale (or quantum) electrochemistry deals with electron motion over molecules’ quantum states through the electron-transfer (ET) rate concept,^16^ based on chemical kinetics and dynamics rather than quantum circuit laws. Accordingly, the ET rate concept was formulated based on the meaning of Arrhenius’s law and transition state theory (TST), the former being an empirical and the latter a semiclassical method that employs both classical and quantum approaches.

The ET rate concept thus has been formulated semiclassically for a ‘wet’ environment where solvent and ions are key components. This theoretical framework is not only pertinent for comprehending biological chemistry, but it also extends to other processes involving the motion of electrons, such as respiration and photosynthesis.^27^ Transitioning from biological to technological applications, the relevance of the ET rate concept becomes particularly apparent in electrochemical devices, such as the *interfacial charge-transfer kinetics* in lithium-ion batteries, which dictates charging/discharging rates, and in *pseudocapacitors*, where surface redox reactions involve electron transfer,^10,28–30^ which are key elements of man-made electronic mobile devices. Here, the urgent task of chemists is to develop better and more efficient energy conversion and storage devices.^31–34^ Notably, these developments are based on the ET rate concept rather than the electron transport that underpins transistor technologies in physics. Thus, while mobile devices—such as smartphones, computers, and some vehicles—depend on semiconductors and processors that utilize nanoscale circuit electronics to function, their unplugged mobility relies critically on the chemical energy stored in batteries and electrochemical capacitors within these devices. This storage technology, in turn, has evolved by relying on the ET rate concept and remains completely separated from the principles of electron transport.

Whether nanoscale circuit electronics avoids ‘wet’ and operates in a ‘dry’ environment, devices based on ET rate concepts strictly depend on electrolytic environments that drive electron motion. For instance, one of the most important concepts involving the ET rate constant is Marcus’s reorganization energy λ_0_ (a Nobel Prize-winning theory) of the solvent.^35–37^ It is λ_0_ that accounts for the slow-rate motion of electrons within ‘wet’ environments, which is in frequencies below 500 Hz, much lower than that of nanoscale electronics that operate above MHz ranges. Marcus’s ET theory departs from TST, as originally formulated in the 1930s by Henry Eyring, which considers the rate constant of chemical reactions as

$$k=\nu_{k}\;\exp\left(-\beta E^{‡}\right)$$ 2

where β = 1/*k*_B_ *T* (*k*_B_ is the Boltzmann’s constant and *T* is the absolute temperature), ν_k_ = κ­(*k*_B_ *T*/*h*), and *E* ^‡^ is the free activation energy of the reaction. In Eyring’s equation, κ is the transmission coefficient, quantifying the probability that a system at the transition state will proceed to products rather than revert to reactants. This coefficient is associated with the electronic coupling between donor *D* (reducers) and acceptor *A* (oxidizers) quantum states (called reactants); see Figure 1(a) for an illustration of what is here referred to as *D* and *A*.

**Figure 1:**
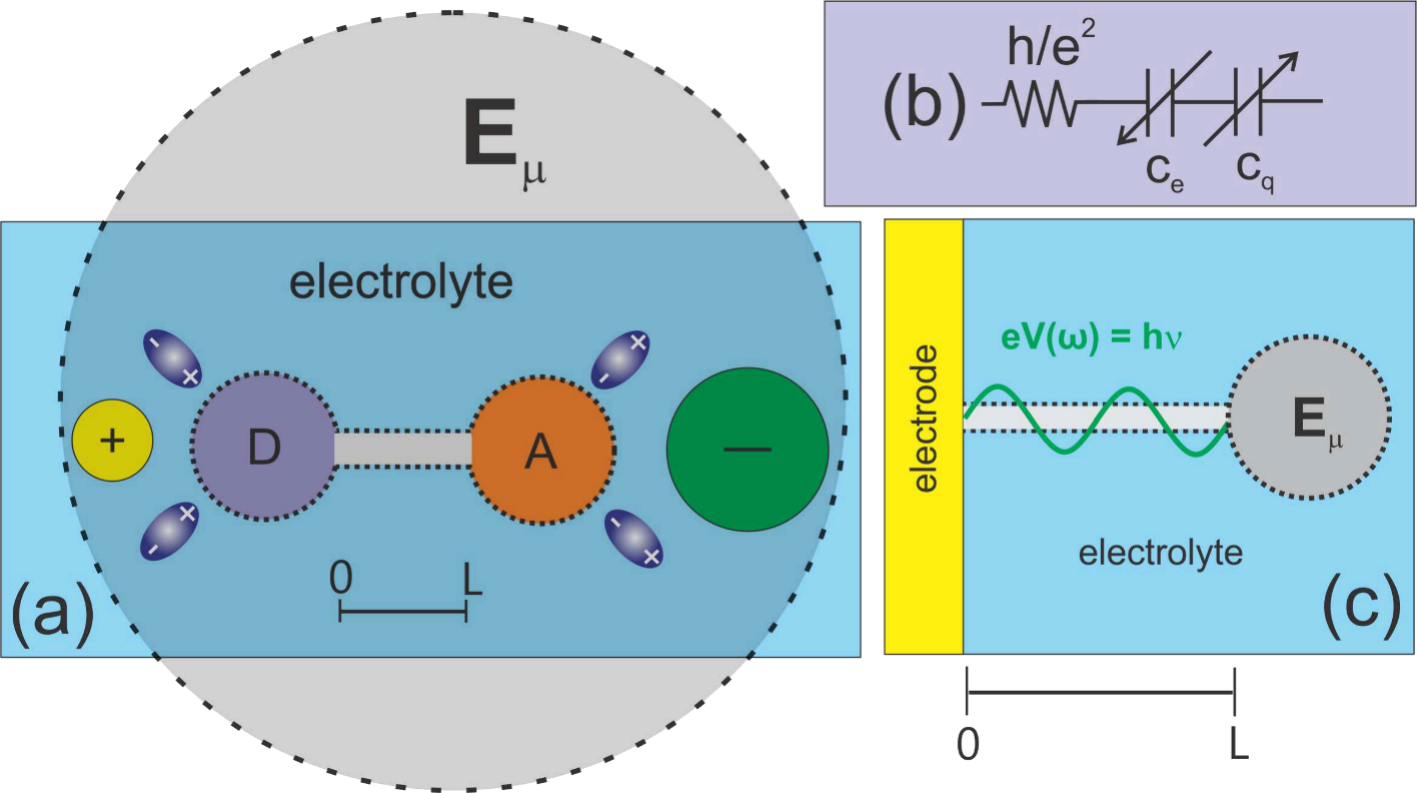
(a) Depictions of *D* and *A* structures with dynamical charge equilibrium involving the electrolyte environment. This equilibrium enables ET transfer between them, consistent with Marcus ET and quantum rate theories discussed in this text. Electron neutrality is maintained for the region around *E*_μ_. (b) Illustrates how the ET dynamics rate, ν_μ_ = 1/τ, is represented through quantum circuit elements. Here, τ = *R*_q_ *C*_μ_. (c) Shows that quantum states and their information become accessible through a time-dependent perturbation. This is expressed as *eV*(ω) = *E*(ω), which causes a modulated, low-energy, photonic-like emission from the electrode with *E* = *hν* (equivalent to *E*(ω) = *ℏω*). This process perturbs the equilibrium charge state of electrochemical state *E*_μ_(ω) = *eV* (ω). The state couples to the electrode via a quantum channel with a length of *L*. The system’s charge relaxation to the perturbation is measured as electric current *i*(ω). The computed impedance or admittance signal can be analyzed in the complex capacitance plane as C*­(ω) ≈ *C*_q_(1 – *jωτ*), as shown further here in section 3. Both *C*_q_ (in the low-frequency limit, ω → 0) and characteristic time τ = *R*_q_ *C*_μ_ are measurable. Thus, the quantum rate is ν_μ_ = 1/τ.

Marcus, considering both Franck–Condon and energy conservation principles simultaneously,^35,37^ defined the meaning of λ_0_ by calculating the ET reaction rate constant through an accurate minimization procedure for an ensemble of configurations of the system that satisfy both Franck–Condon and energy conservation principles constraints. This approach results in an activation energy of *E* ^‡^ = (*E* ^0^ + λ_0_)^2^/4λ_0_, where *E* ^0^ is the standard free energy of the electrochemical reaction, and λ_0_ is the fundamental component of Marcus theory that considers the role of the ‘wet’ dynamics during the ET reaction proceeding through eq 2. Motivated by this framework, a long-standing question is whether *G* (eq 1) and *k* (eq 2) can be unified through a first-principles quantum mechanical analysis that integrates both concepts.^19,20,38–43^ To address this question, we present a theoretical approach.

## 2 Quantum-Rate Theory

QR theory defines a fundamental quantum frequency ν as the ratio between the reciprocal of the von Klitzing constant *R*_K_ = *h*/*e* ^2^ (≈ 25.8 kΩ) and the quantum capacitance *C*_q_ such as^2^

$$\nu =\frac{e^{2}}{hC_{q}}=\frac{E}{h}$$ 3

where *E* = *e* ^2^/*C*_q_ is the energy associated with the quantum capacitance state. This energy is the charging energy required to add a single electron to the quantum state, making *E* proportional to the chemical potential difference Δμ between the *D* and *A* states [see Figure 2(a)]. The quantum capacitance *C*_q_ is directly related to the electronic density-of-states (DOS) of the material by *C*_q_ = *e* ^2^(d*n*/d*E*). Equation 3 thus expresses the fundamental quantum relationship where the frequency (ν) of a process is proportional to the energy (*E*), linked by Planck’s constant (*h*). Nevertheless, how do the dynamics within eq 3 govern the low-energy dynamics of electronics (operating in an electrolyte environment) and ET reactions?

**Figure 2:**
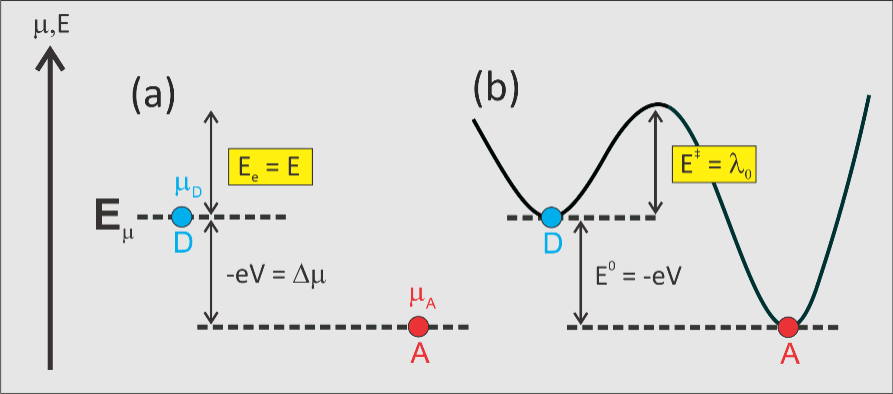
(a) A quantum representation shows adiabatic electron conductance through *D* (blue) and *A* (red) states, with *E* ^0^ = −*eV* as described in (b). Here, Δμ = μ_D_ – μ_A_ refers to the difference in electrochemical potentials between *D* and *A*, equal to *e* ^2^/*C*_q_. This energy links to electron current *i* = −*e*(c_*_/*L*)­Δμ­(d*n*/d*E*), where quantum channel length *L* is captured by the density-of-states (d*n*/d*E*) = *g*_s_ *L*/c_*_ *h*, defining electron transport via *L*. (b) Marcus’s ET diagram, linked to (a), applies when *E* ^0^ equals −λ_0_; in magnitude (ignoring thermodynamics), this means *E* ^‡^ ≈ λ_0_.

To address the above question, we generalize the concept in eq 3 by expressing the total quantum conductance *G* (from Landauer’s formula, eq 1) in terms of its inverse, the quantum resistance *R*_q_ = 1/*G*, serving as the replacement for *R*_K_. The electrochemical capacitance *C*_μ_ similarly replaces *C*_q_. The generalized quantum rate ν_μ_ is defined as the inverse of the system’s characteristic electrochemical relaxation time τ, with τ = *R*_q_ *C*_μ_. Thus, ν_μ_ = 1/τ = *G*/*C*_μ_.

The total quantum conductance *G* is given by *G* = *G*_0_ ∑_*n*_^*N*^ *T*_*n*_. The electrochemical capacitance *C*_μ_ results from the series combination of the classical electrolyte capacitance *C*_e_ (which accounts for spatial-Coulombic charge separation, or polarization) and the quantum capacitance *C*_q_:

$$\frac{1}{C_{\mu }}=\frac{1}{C_{e}}+\frac{1}{C_{q}}$$

By substituting these definitions into ν_μ_ = *G*/*C*_μ_, we obtain

$$\nu_{\mu }=\frac{G}{C_{\mu }}=G_{0}\left(\overset{N}{\underset{n}{\sum }}T_{n}\right)\left(\frac{1}{C_{e}}+\frac{1}{C_{q}}\right)=\frac{g_{s}}{h}\left(\overset{N}{\underset{n}{\sum }}T_{n}\right)E_{\mu }$$ 4

where the last equality follows from substituting *G*_0_ = *g*_s_ *e* ^2^/*h* and recognizing that the reciprocal of the total capacitance 1/*C*_μ_ yields the electrochemical energy *E*_μ_ = *e* ^2^/*C*_μ_. Note that *C*_μ_ is an equivalent capacitance 1/*C*_μ_ = 1/*C*_e_ + 1/*C*_q_ [refers to Figure 1(b)], i.e., the circuital series combination of *C*_e_, the spatial-Coulombic charge separation (also referred to as polarization), and *C*_q_. The total energy of this homogeneous system comprises *D* and *A* moieties and their electrolyte surrounding, as shown in Figure 1(a), corresponds to *E*_μ_ = *e* ^2^/*C*_μ_ [that can also be perturbed by a low-energy ‘photoemission’ perturbation from the electrode, as shown in Figure 1(c)].

The meaning of *C*_μ_ is particularly important for physicochemical situations.^28,44,45^ In these cases, the energy associated with the electrolyte’s charge dynamics, *E*_e_ = *e* ^2^/*C*_e_, is in dynamical equilibrium with the electronic energy, *E* = *e* ^2^/*C*_q_. This implies that the *C*_e_ component is equivalent to *C*_q_, i.e., *C*_e_ ≈ *C*_q_. Experiments and computational simulations confirm this.^46^ In this case, an electric degeneracy of *g*_e_ = 2 can be assigned. The total (equivalent) energy is then established as *E*_μ_ = *e* ^2^/*C*_μ_ ≈ *g*_e_(*e* ^2^/*C*_q_) = *g*_e_(*e* ^2^/*C*_e_). Here, *E*_e_ = *e* ^2^/*C*_e_ represents the ‘wet’ environmental contribution, which matches the magnitude of the quantum mechanical states (electronic structure) of the reactants, *E* = *e* ^2^/*C*_q_.

Given this dynamical equilibrium constraint, Marcus’s ET rate constant in eq 2 can be shown to be a specific case of the QR theory (see more details of the elementary mathematical steps in the Supporting Information). This is demonstrated by applying statistical mechanics to the spin-degenerated and nonadiabatic quantum electrochemical energy state defined in eq 4. This provides an electrochemical generalization of the basic assumptions in eq 3.

Thereupon, the occupancy of the energy state, where *E* of a quantum state in the grand canonical ensemble is related to the occupancy *f* by the Fermi–Dirac distribution, which for a single electron transfer (*N* = 1) is *f* = [1 + exp­(*βE*)]^−1^. To generalize the electrochemical rate ν_μ_ to account for the full thermal broadening of the quantum states, the thermalized quantum rate ν_μ_(*T*) is defined by recognizing that the highest probability of an ET event occurs where the probability of the donor state being occupied (*f*) and the acceptor state being available (1 – *f*) is maximized. This product, *f*(1 – *f*), describes the available density of states for transfer at the temperature *T*.

The classical TST framework assumes that the rate is proportional to the thermal energy *k*_B_ *T*. By factoring out the *k*_B_ *T*/*h* dependence from ν_μ_, and including the degeneracy *g*_e_, the full quantum-thermal rate ν_μ_(*T*) is written as

$$\nu_{\mu }\left(T\right)=g_{e}\frac{h}{k_{B}T}\cdot \nu_{k}\cdot \frac{1}{\beta k_{B}T}f\left(E\right)\left[1-f\left(E\right)\right]$$ 5

where the factors *h*/*k*_B_ *T* and (*βk*_B_ *T*)^−1^ are dimensional normalization terms that cancel out (since β = 1/*k*_B_ *T*), leaving ν_μ_(*T*) proportional to ν_k_ and the occupation product *f*(1 – *f*). This formulation ensures consistency with the Landauer conductance formalism while utilizing the TST prefactor ν_k_ for comparison. The semiclassical limit (Marcus ET) is recovered by applying the Boltzmann approximation to eq 5.

For highly activated processes, corresponding to the semiclassical limit of Marcus’s theory, the states are sparsely occupied, meaning *βE* ≫ 1. This condition yields *f* ≈ exp­(−*βE*) and 1 – *f* ≈ 1. Substituting these into eq 5 and setting *g*_e_ = 1 (disregarding degeneracy), the rate becomes

$$\nu_{\mu }\left(T\right)\approx \nu_{k}\cdot f\approx \nu_{k}\;\exp\left(-\beta E\right)$$ 6

This result, ν_μ_(*T*) = ν_k_ exp­(−*βE*), is the semiclassical limit and is equivalent to the fundamental kinetic expression predicted by Marcus’s ET theory, where *E* is identified as the activation energy *E* ^‡^ (which is better demonstrated below). This confirms that the semiclassical Marcus ET rate is a particular setting (the Boltzmann limit) of the more general quantum mechanical rate description provided by eq 4.

An alternative deduction of the rate can be made by explicitly expressing it in terms of *C*_q_, and by recognizing the thermodynamic definition of quantum capacitance as *C*_q_ = *e* ^2^(d*n*/d*E*). The thermal derivative of the occupation number for a single state is given by (d*n*/d*E*) = β­[*f*(1 – *f*)]. By identifying the quantum rate through the relation ν_μ_ ∝ *E*/*h*, and substituting the thermal energy, *E* = *e* ^2^/*C*_q_, which leads to *E* = 1/(β­[*f*(1 – *f*)]), the resulting expression is then scaled by the degeneracy factors (*g*_s_ *g*_e_) and the TST prefactor ν_k_. This yields

$$\nu_{\mu }=g_{s}g_{e}\nu_{k}{\left[f\left(1-f\right)\right]}^{-1}$$ 7

This expression shows the inverse relationship between the rate and thermal broadening of the state. To compare directly with the TST framework, we substitute the classical pre-exponential factor definition, where ν_k_ = κ­(*k*_B_ *T*/*h*), with κ equal to the sum over transmission probabilities, i.e., κ = ∑_*n*_^*N*^ *T*_*n*_, into the quantum rate analysis. This highlights a core principle: in electrochemistry, slower kinetic rates arise from thermal broadening of quantum states and the environmental dielectric contribution, *C*_e_. In the QR formalism, this effect is quantified by the thermally derived *C*_q_, which combines with *C*_e_ to determine the rate constant.

Nonetheless, a final step is required to fully compare the rate *k* = ν_k_ exp­(−*βE* ^‡^), as originally proposed by Marcus, with the consideration of *E* ^‡^ = (*E* ^0^ + λ_0_)^2^/4λ_0_ into eq 2, and the rate ν_μ_ = ν_k_ exp­(−*βE*) obtained from eq 5, formulated based on premises of the QR theory. Namely, this pace involves recognizing for which physical conditions, *E* ^‡^, defined in eq 2 as a function of λ_0_, equates to *E*.

Mathematically, it can be noted that whether *E* ^0^ (the standard thermodynamic driving force of the reaction) has the same magnitude as λ_0_ (the reorganization energy from the solvent), then *E* ^‡^ equates to *E* ^0^ = λ [see Figure 2(b)]. In the QR theory, this is equivalent to equating the magnitude of *E*_e_ (the electrolyte’s charge energy contribution) to *E* (the energy difference between quantum electronic states of the donor *D* and acceptor *A*), for which *E*_μ_ (the grand potential^47,48^ associated with the ET reaction) equates to *g*_e_ *E* = *g*_e_ *E*_e_, where *g*_e_ is the energy ‘degeneracy’ between quantum and classical states. This implies that *E* = *E*_e_ and *E* = *E*_e_ = *E*_μ_/*g*_e_ is the chemical potential of the ET reaction, considering both internal (electronic) and external (electrolyte charge) energy contributions [see Figure 2(a)]. This scenario, where *E* = *E*_μ_/2, is the condition in which both theories are comparable, i.e., *E* = *E*_e_ is the QR equivalent of *E* ^0^ = λ_0_ in Marcus’ theory. Thus, the mathematical analysis is equivalent in each theory, with λ_0_ and *E*_e_ representing solvent or electrolyte effects, and *E* ^0^ and *E* representing the electronic contributions to the ET reaction.

As *E*_μ_ is the grand potential, the thermodynamics and charge neutrality conditions must be considered. Electrolyte particles shield the electronic *D* and *A* states. The previous mathematical assumption, which only compared the magnitudes of the energy variables, overlooked these physical realities. The correct physical picture is described by −*E* ^0^ ≈ λ_0_ [see Figure 2(b)]. Here, the electrolyte acts as a thermal and ionic charge reservoir that drives the electrodynamics. For this case, *E* ^‡^ is null, which implies a ‘barrierless’ ET. In Marcus’s ET viewpoint, this means reactants become products without overcoming a free energy hurdle. From a quantum perspective, rather than classical kinetics, the ET reaction proceeds as a tunneling process. QR theory shows that, beyond being quantum tunneling,^49^ this process is coherent, with tunneling probability proportional to *g*_s_ *e* ^2^/*h* ≈ 77.5 μS (see below).^44,45^

This is the optimal rate, corresponding to the maximum possible rate, and, in the terminology of chemical kinetics, the reaction is considered ‘activationless.’ In the classical parabolic curve representation of the *D* and *A* states (see Figure 2), this situation marks the limit at which the reaction can proceed before entering the Marcus inverted region. In summary, this condition represents a ‘Goldilocks’ zone for ET reactions, where the driving force required for the reaction is perfectly balanced by electrolyte dynamics (in Marcus’s ET analysis, this dynamic is referred to as the solvent’s reorganization energy). This balance leads to the fastest reaction rate.

In a QR analysis,^2^ this condition corresponds to electron conductance through *D* and *A* states as a wave (transmittance), where Δμ = *e* ^2^/*C*_q_ = −*eV* = *E* and *E* represents the chemical potential difference between *D* and *A* states. The energy Δμ = −*eV* ∝ *e* ^2^/*C*_q_ required for the ET reaction links to an electric current *i* = −*e*(c_*_/*L*)­Δμ­(d*n*/d*E*) through a quantum channel between *D* and *A* states, as shown in Figure 2(a). This quantum channel [Figure 1(a)] has a length *L*, measured through the density of states (d*n*/d*E*) = *g*_s_ *L*/c_*_ *h*, which defines transport through *L*. Since Δμ = −*eV* [see Figure 2(a)], the ratio *i* to *V* = −Δμ/*e* gives the conductance quantum *G* = *i*/*V* = *g*_s_ *e* ^2^/*h* = *G*_0_, corresponding to quantum coherent transport in the ET reaction. Alternatively, since *C*_q_ = *e* ^2^(d*n*/d*E*), the quantum rate ν is ν = *G*_0_/*C*_q_ = c_*_/*L* = *g*_*s*_(*e* ^2^/*C*_*q*_)*h* = *g*_s_(*E*/*h*). It is important to anticipate that this quantum-rate ν = *G*_0_/*C*_q_ framework are supported by experimental characterizations, including structural analysis (Figure 3) and electrochemical impedance spectroscopy (Figure 4).

**Figure 3:**
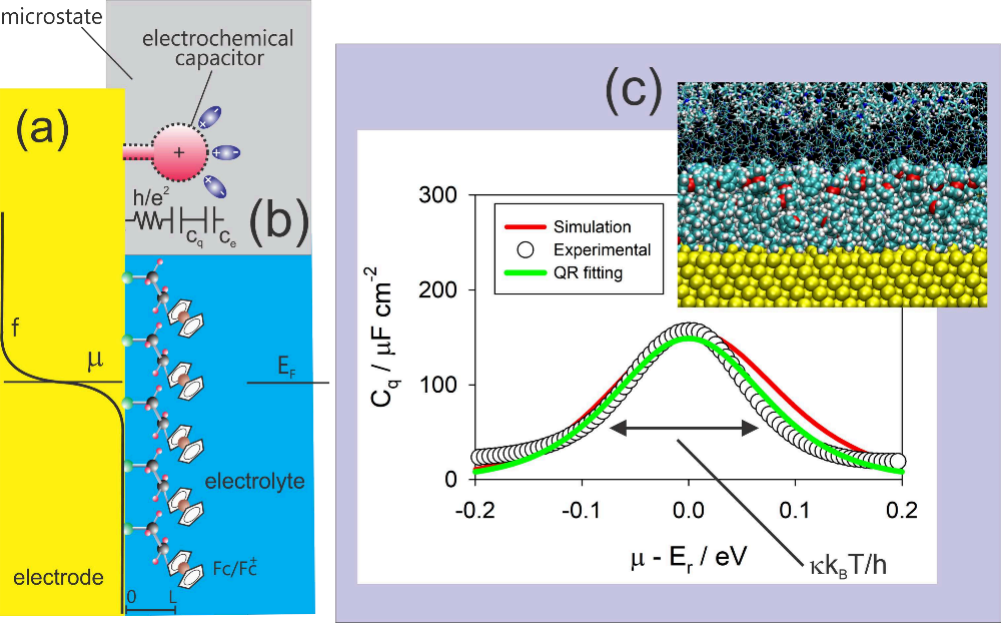
Panels (a) and (b) show a molecular ensemble of redox switches^50^—a type of molecular system capable of reversible electron transfer reactions—arranged over a metallic electrode, with each redox switch represented by a quantum resistance-capacitance (RC) circuit element,^2,55^ which models quantum transport and storage of charge. In (a), μ refers to the chemical potential, which represents the energy at which electrons reside in the electrode, while *E*_F_ is the Fermi-level (highest occupied energy level at absolute zero) of the molecular film (ensemble), also known in electrochemistry as the formal potential of the redox switch film, indicating its tendency to gain or lose electrons. Panel (c) shows finite-temperature experimental data (dot circles) for *C*_q_, the quantum capacitance, which quantifies the system’s ability to store energy at the quantum level, following *C*_q_ = *Ne* ^2^/(*k*_B_ *T*)*f*(1 – *f*) (green line). This agreement supports the quantum redox (QR) theory. The computational simulation (red line) predicts the occupancy of states that exhibit both quantum and classical characteristics and also matches the experimental data. The inset displays a computational model cluster, including gold atoms from the electrode used in the analysis. Note that the width of the density of states (DOS) depicted in this figure relates to *κk*_B_ *T*/*h* in QR theory. Panels (a) and (b) are reproduced (adapted) from ref 46 with permission from Paulo R. Bueno. Copyright (2020) Royal Society of Chemistry. Reproduced (adapted) from ref 43 with permission from Paulo R. Bueno, Vinícius W. D. Cruzeiro, Adrian E. Roitberg, and Gustavo T. Feliciano. Copyright (2021) Elsevier.

**Figure 4:**
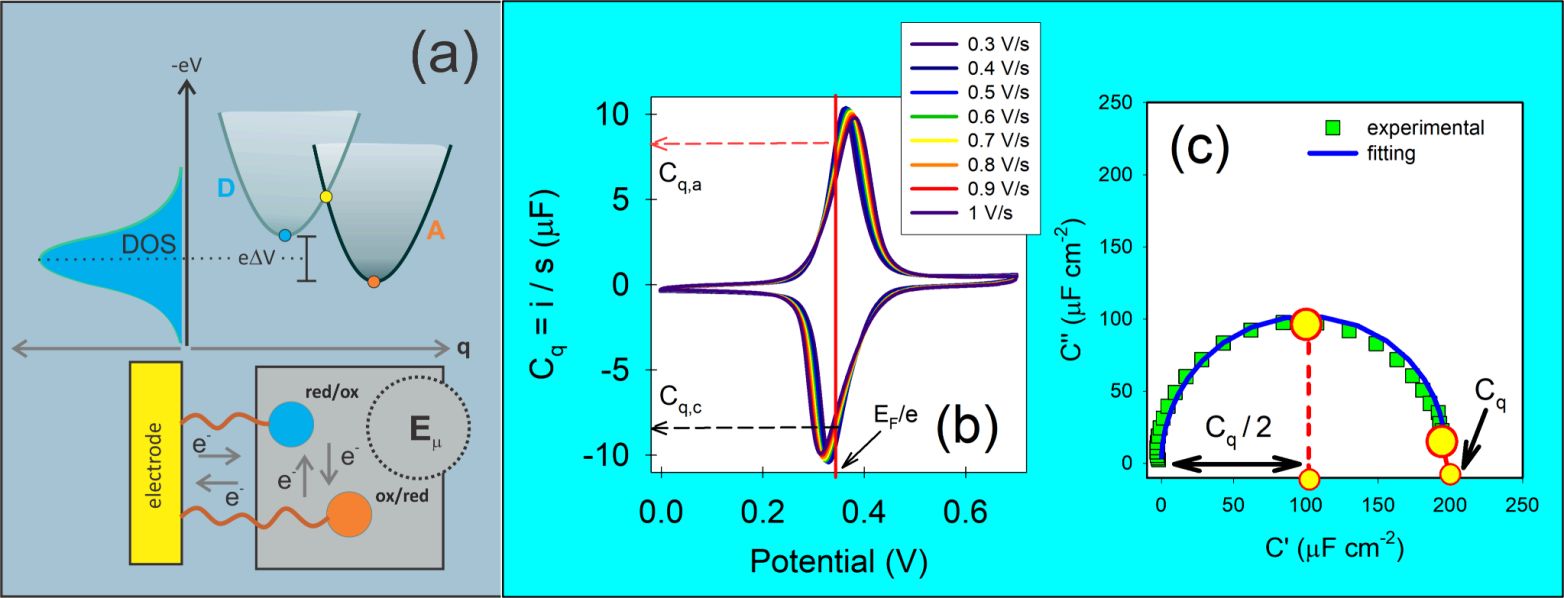
(a) Schematic representation of the energy states and electrodynamics of a redox switch monolayer with an electrode. The *e*Δ*V* energy state of the electrode corresponds to that of the Fermi level *E*_F_/*e* of the monolayer, as indicated in (b). This is an equilibrium state between the chemical potential of the electrode and the formal potential of the monolayer. *E*_μ_ represents the energy of an ensemble of individual microstates depicted in Figure 1(a) with a DOS given by *C*_μ_/*e* ^2^, that are accessed through a perturbation of the electrode as exemplified in Figure 1(b). (b) Corresponds to the scan rate *s* normalized *i*/*s* = *C*_q_ electric current *i* versus potential curves of a redox switch monolayer assembled on a gold electrode for different values of *s*, ranging from 0.3 V s^–1^ to 1 V s^–1^. As noted, the value *C*_q_ is constant across different *s*, demonstrating the robustness of QR theory. The values of *C*_q_ can be obtained at either the anodic *C*_q,a_ or cathodic *C*_q,c_ current regions of this normalized CV plot. (c) Capacitive Nyquist spectrum obtained at the formal potential *E*_F_/*e* of the electrode, as indicated in (a). According to C*­(ω) ≈ *C*_q_(1 – *jωτ*), *C*_q_ can be obtained from this diagram as the diameter of the semicircle (where ω → 0), i.e., as ∼200 μF cm^–2^. Reproduced (adapted) from ref 44 with permission from Erika V. G. Alarcón, Adriano Santos, and Paulo R. Bueno. Copyright (2021) Elsevier.

However, where does the energy required for electron transfer (ET) and coherent transport originate? According to statistical mechanics, in order to achieve equilibrium dynamics between *E* = −*eV* = Δμ and the electrolyte *E*_e_, the grand potential *E*_μ_ = *E*_e_ + *E* must be zero. This condition requires that *E* = −*E*_e_, demonstrating that the energy for ET is derived from the electrochemical potential difference between the system and the electrolyte. This result aligns with the earlier discussion of the QR formulation of Marcus’s ET analysis, where an additional *g*_e_ degeneracy is needed to describe the rate dynamics.

To conclude so far, the situation in which *E* ^‡^ = 0 (−*E* ^0^ = λ_0_), as discussed above, achieved from a semiclassical analysis, is equivalent to that of the QR theoretical viewpoint, in which *E*_μ_ = 0 (−*E* = *E*_e_). The latter corresponds to the situation in which the environment electrolyte charge dynamics provides an amount of energy equivalent to Δμ = −*eV* ∝ *e* ^2^/*C*_q_ that permits the ET reaction to proceed coherently. However, there are important physical differences between semiclassical and quantum rate analysis of ET dynamics; i.e., the QR theory predicts a quantum coherent transport of electrons, where the resistance for ET to occur is *R*_q_ = *h*/*g*_s_ *e* ^2^ ≈ 12.9 kΩ, which has been demonstrated experimentally^44,45^ (see examples in the next sections).

It is essential to clarify what is meant by ‘quantum coherence’ in this context. According to quantum electronics, this refers to a configuration where the characteristic length *L* of the quantum channel between *D* and *A* moieties is less than the phase coherence length *L*_ϕ_ of the system. For example, when −*V* = Δμ/*e* = *e*/*C*_q_ drops to zero in the quantum channel, an external +*V* = *e*/*C*_e_ potential must be provided by the electrolyte. This setup requires equilibrium dynamics between the electron in the quantum conductor channel and charged particles in the electrolyte.

For quantum coherence, the delocalized electronic wave function of the electron reaches an energy uncertainty that equates to Δμ = *e* ^2^/*C*_q_ = *E*, which, according to Heisenberg uncertainty principles, has a lifetime that corresponds to τ ∼ *R*_q_ *C*_q_ ∼ (*h*/*e* ^2^)*C*_q_ [a circuit representation of this lifetime is shown in Figure 1(b)]. Considering the external (electrolyte) environment contribution, this directly correlates to the electrochemical rate of eq 4 such that ν_μ_ = 1/τ, where τ = *R*_q_ *C*_μ_. To compare τ = *R*_q_ *C*_μ_ to τ = *R*_q_ *C*_q_, this requires an uncertainty in the measuring of the total energy *E*_μ_ = *g*_e_(*e* ^2^/*C*_q_), which increases by *g*_e_ owing to *C*_e_ ≈ *C*_q_ (*g*_e_ = 2), leading to a higher value than that hypothetical situation in which the electrolyte does not contribute to the transport dynamics. Hence, the analysis is in agreement with the uncertainty principle. Namely, the quantum mechanical coherence can be maintained despite the electrolyte’s dynamics controlling the rate, as it is the slowest process; despite being stochastic, it is coherent (a condition not so unusual to mesoscopic systems).

To model nonadiabatic processes in QR theory, the channel’s transmission probability, or ∑_*n*_^*N*^ *T*_*n*_, is less than 1. For simple tunneling, this equals κ = exp­(−*γL*), where γ is the decay constant. Tunnel coherence can be studied by measuring the frequency-dependent conductance *G* (see the next section), a macroscopic property. This conductance is related to the quantum mechanical tunneling probability *T*. Since electrons act as waves scattering in the potential barrier between *D* and *A*, *G* depends on their likelihood to cross the barrier, revealing coherence. If present, this indicates that ET occurs without a loss of phase, even in the presence of environmental entropy resulting from electrolyte charge dynamics.

Ultimately, QR theory encompasses Marcus’s ET theory as a specific case^1,2,50^ and incorporates electron transport as a fundamental component of the theory. The connection between electron transport *G* and rate *k* is given by *C*_μ_, i.e., *G* = *kC*_μ_. This straightforward link unites two historically separate concepts, providing a more comprehensive view of electron transport and ET processes in electrolytes. Because QR theory relates to measurable circuit elements (*R*_q_ and *C*_μ_) that correlate with the energy occupancy of states *E*_μ_, it allows simpler experimental investigation of various phenomena, some of which are discussed below.

## 3 Examples of Quantum Coherence in ‘Wet’ Ambient: Applications to Functional Material Interfaces

For a comprehensive understanding of the different applications in which QR theoretical concepts demonstrate their usefulness, this section is divided into three parts. First, it presents the case in which a redox-active switch monolayer system^44,45^ is studied using both experimental^44,45^ and computational approaches.^46^ Next, it discusses how these concepts apply to revealing the respiration process of Geobacter.^3^ Finally, it illustrates, in the absence of redox reaction processes, how the electronic structure of quantum dots^51^ and graphene^52^ can be investigated by employing the coherent transport provided by the theory as an *in situ* method for calculating the electronic structures of these nanoscale materials.

### 3.1 Redox Switches

A well-defined self-assembly monolayer containing redox probes constitutes a typical redox-switch interface.^53,54^ The redox reaction within these monolayers can ‘resonate’ with the states of the electrode, as shown in Figures 3(a) and 4(a). Owing to this ‘resonance’, a finite-temperature redox DOS is measurable as *C*_q_ = *Ne* ^2^/(*k*_B_ *T*)*f*(1 – *f*). This is depicted in Figure 3 and Figure 4(a). The occupancy of *C*_q_ is mapped by scanning the electrode’s (electro)­chemical potential using time-dependent electrochemical methods such as impedance spectroscopy.^2,45^ The redox DOS (*C*_q_/*e* ^2^) is accessible by measuring the value of *C*_q_ at the low frequency limit as a function of the electrode’s potential.^2^ For lower-frequency dynamics, a dynamical charge equilibrium exists between *E*_e_ and *E* states, as discussed previously within QR theory. A computational method was used to simulate the charge state conditions of the monolayer in the presence of the experimental electrolyte. This method confirmed that charge equilibrium dynamics operates, as shown in Figure 3.

Note that this computational approach^46^ used statistical, not quantum, mechanics. This allowed us to conclude that, since charge dynamics reach equilibrium as *E*_e_ = *E* ∝ *e* ^2^/*C*_q_, we could compute the state of charge and the electronic density distribution that determines the system’s grand potential *E*_μ_,^47,48^ which contains quantum information. Figure 3 shows good agreement between experimental data, QR fitting to *C*_q_ = *Ne* ^2^/(*k*_B_ *T*)*f*(1 – *f*), and simulated *C*_q_ results, supporting the QR premises.^46^ Specifically, *E*_e_ = *E* ∝ *e* ^2^/*C*_q_ was calculated as the state of charge in equilibrium with the electrolyte’s charge dynamics, corresponding to minimal energy. This state, in which the electronic density distribution is given by the system’s grand potential *E*_μ_,^47,48^ shows that the linear perturbation and relaxation of this energy and charge equilibrium (with the electrolyte) contains quantum information, which can be obtained if the relaxation time is computed.

Additionally, it is owing to *E*_μ_ = *e* ^2^/*C*_μ_ = *g*_e_(*e* ^2^/*C*_q_), i.e., *C*_q_ ≈ *C*_e_, that we obtain the rate dynamics from electrochemical current–potential curves.^44^ This is exemplified in Figure 4(b), where anodic *C*_q,a_ or cathodic *C*_q,a_ current-derived values estimate the *C*_q_ value of the interface. This yields an average value over all possible accessible states of approximately 8.5 μF, and the rate was thus estimated to be this value. For instance, because *C*_μ_ = *q*/*V*, energy is stored within an electric and dynamical current: *i* = *q*/τ = *C*_μ_(*V*_μ_/τ). Here, *V*_μ_/τ is intrinsic to the system but can be perturbed by an external scan rate *s* = d*V*/d*t* applied to the system, i.e., *i* = *C*_μ_ *s*.^44^ This establishes a direct relationship between *i* and *s* through the meaning of *C*_μ_. From this, the rate ν_μ_ = 1/τ = *g*_e_/*R*_q_ *C*_q_ can be estimated accurately by applying eq 4, from which ν_μ_ = *g*_e_ *G*_0_/*C*_q_ is obtained. Noting that *g*_e_ *G*_0_ ≈ 155 μS and *C*_q_ ≈ 8.5 μF, a ν_μ_ of ≈18 Hz is attained.^44^ This agrees within experimental error with those obtained by traditional methodologies, based on semiclassical Butler–Volmer analysis. For example, the method proposed by Laviron^56^ estimates the ET rate as ≈17 Hz.^45^

An alternative, more accurate than the *i* versus *V* approach, is to employ a time-dependent method that provides access to a complex capacitance function, C*­(ω) ≈ *C*_q_(1 – *jωτ*). Here, ω is the angular frequency and τ = 1/ν_μ_ is the relaxation characteristic time directly correlated to the electrochemical rate.^44^ As illustrated in Figure 4(c), *C*_q_ is obtained by directly analyzing the C*­(ω) = *C′* + *jC*″ spectrum at low frequencies; for ω → 0, this yields the limiting value of *C*_q_, which marks the end point of the capacitive Nyquist (*C*″ versus *C′*) plot. This *C*_q_ value is about 200 μF cm^–2^. For an average electroactive electrode area of about 0.044 cm^–2^, this corresponds to 8.8 μF, yielding a ν_μ_ of about 17.6 Hz.^44^ Within experimental errors, this result matches that obtained by current–potential methods [Figure 4(b)].

Let us clarify the above analysis in the context of that introduced in section 2. Here, we focus on an analysis of the quantum rate based on a single electron. The single electron approach also applies to a redox switch monolayer within a two-dimensional electron gas structure,^2,57^ as shown in Figure 4(a). Each redox switch molecule is an individual moiety contributing to one quantum RC state in the ensemble. Namely, each donor–acceptor (D-A) moiety within the monolayer operates as a microstate.^50^ It represents an individual quantum-resistive-capacitive (RC) element in an ensemble of parallel quantum RC circuits attached to the electrode (see, for instance, Figure 3(a) for the case of a redox switch film). This configuration serves as a microscopic probe for the ensemble of individual single quantum RC states.^50^ For the expression ν_μ_ = 1/τ = *g*_e_/*R*_q_ *C*_q_, the measurable quantum capacitance *C*_q_ (determined by current–potential curves or time-dependent methods) equals *Nc*_q,i_. Here, *c*_q,i_ denotes the capacitance of each molecular redox entity attached to the electrode. Similarly, for *G* = 1/*R*_q_, the total conductance of the ensemble is *G* = *NG*_0_κ. Thus, ν_μ_ = *g*_e_ *g*_s_ *N*(*e* ^2^/*h*)­κ/*C*_q_ = *g*_e_ *g*_s_(*e* ^2^/*h*)­κ/*c*_q,i_ quantifies the average ν_μ_ for a single state within the ensemble. This applies when the constant 2*G*_0_ is divided by the total ensemble capacitance *C*_q_. This relationship is illustrated in the analysis of Figure 4(b) and (c).

Regarding charge relaxation in coherent quantum systems, the QR theory, introduced in section 2, describes quantum mechanics within chemical kinetics and includes the semiclassical ET theory as a special case.^2^ This theory aligns with the quantum coherence predicted by the mesoscopic physics (‘dry’ environment) theory by Büttiker^58^ and validated by Gabelli.^59^ The QR theory, developed to quantify the ET rate constant at room temperature in an electrolytic (‘wet’) medium, as briefly reviewed in Section 2, is consistent with charge relaxation dynamics observed in coherent mesoscopic (‘dry’) systems at low temperatures and in a vacuum.^59^

A comparison between ‘dry’ and ‘wet’ quantum coherent systems can be made by examining the region encompassing *E*_μ_ within the redox switch state [as shown in both Figures 1(a) and 4(a)], which acts as a scattering region. In this region, the energy states of the microscopic entity influence electron transport, as described in the ‘dry’ mesoscopic physics theory of electron behavior in small-scale systems. The charge accumulated in this scattering region is given by *q*(*t*) = (1/*h*)∑_*i*_^*N*^ ∫ τ_*i*_(*E*)*f*(*E*, μ_*i*_(*t*))­d*E*, where *i* labels individual quantum RC (redox-active) states (modeled as individual quantum RC circuits, referring to electronic models that capture charge storage and flow at the quantum scale^50^).

In Büttiker’s work,^58^ *i* is referred to as α, which corresponds to one of two individual electron reservoirs (leads) connected to a mesoscopic capacitor—the other reservoir is labeled β. In this context, β corresponds to the electrolyte serving as a gate in the electrochemical setup, as depicted in Figure 1. Moving from this physical setup to the system’s frequency response, the complex admittance at the low frequency limit is given by G*­(ω) = jωC*­(ω) ≈ *jωC*_q_ + (*ωC*_q_)^2^ *R*_q_. Here, the real component Re­[G*­(ω)] = (*ωC*_q_)^2^ *R*_q_ of this expression corresponds to the conductance. This conductance, which manifests as a *dissipative electric current*, is a key signature of the coherent charge relaxation associated with quantum regime dynamics. Notably, the *R*_q_ term, along with *C*_q_, is measured by a frequency response analyzer (FRA) in potentiostat equipment at a defined low-frequency limit. This specific frequency corresponds to where the semicircle in Figure 4(c) ends. By combining the analysis of C*­(ω) (from which *C*_q_ is precisely obtained) with that of G*­(ω), it becomes apparent that in a coherent quantum regime, the conductance is not simply a real number, as in the direct-current (DC) Landauer formula (eq 1), but rather a complex function of frequency that encodes both the quantum capacitance *C*_q_ and the charge relaxation resistance *R*_q_.

The QR theory is characterized by the indissoluble correlation between *R*_q_ and *C*_q_, which governs the rate of redox reactions and electronics in electrolyte ambient.^2,28,50,60^ In particular, the charge transfer relaxation *R*_q_, dictating the rate of charge injection within *E*_μ_ states, is linked to the fluctuations of electrochemical states in the electrolyte. Thus, it is the electrolyte dynamics that controls the overall time τ = *R*_q_ *C*_μ_. The coefficient ω^2^ of the real part of G*­(ω), representing *R*_q_ *C*_q_^2^, can be directly measured in electrolyte environments. Notably, a measurable value of *R*_q_ = *h*/2*e* ^2^ ≈ 12.9 kΩ corresponds to quantum coherent charge relaxation. Observing this quantum coherent phenomenon at room temperature for redox reactions and electronics within an electrolyte environment (see the further examples below) marks a significant achievement of QR theory. This result has significant implications for designing molecular systems and nanoscale moieties that can operate quantum computations at room temperature.

Accordingly, the consistency of the QR theoretical analysis is also verifiable by evaluating the total conductance *G*. According to eq 4, *G* = *G*_0_ ∑_*n*_^*N*^ *T*_*n*_ = *G*_0_ *Nκ*. Experimentally, the value of *N* was independently estimated from the experimental equilibrium DOS [see Figure 5(a)]. This value corresponds to the integral over all potential energy states of the DOS, as 6.3 × 10^12^ states.^45^ The tunneling property, given by κ = exp­(−*γL*), was estimated by considering a thickness of *L* of 1.8 nm for the redox switch film. This thickness was estimated from ellipsometry measurements. A γ of approximately 1.25 Å^–1^ was taken from the literature data. Using these values resulted in *G*_0_ = *G*/*Nκ* ≈ 12.9 kΩ, as predicted by eq 4.^45^

**Figure 5:**
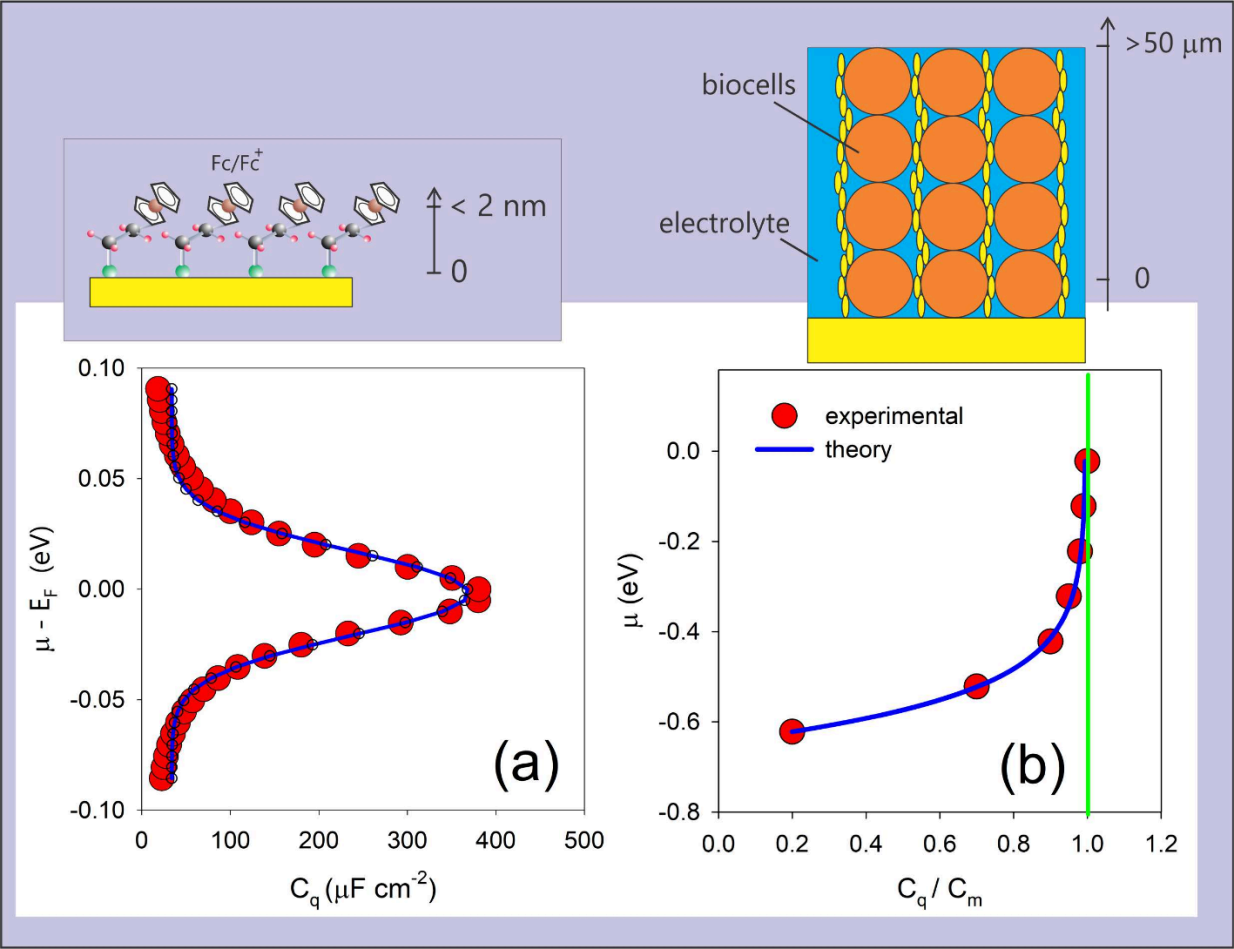
(a) The experimental DOS shapes of redox-active, and (b) biological films, both agree with *C*_q_ = *Ne* ^2^/(*k*_B_ *T*)*f*(1 – *f*) (blue line). For (b), *C*_q_ = *Ne* ^2^/(*k*_B_ *T*)*f*(1 – *f*) is set to approximately *C*_m_ exp­(*eV*/*k*_B_ *T*), where *f* (the Fermi–Dirac occupancy) is approximated by a Boltzmann occupancy. The insets in panels (a) and (b) show the short- and long-range lengths *L* for the electron transfer (a) and transport (b) cases. This figure is reproduced (adapted) from ref 3 with permission from Paulo R. Bueno. Copyright (2024) Royal Society of Chemistry.

An equivalent circuit analysis of the interface, where *R*_q_ = 1/*G*_0_, demonstrates that this relaxation quantum mechanical coherent resistance reflects the total series contribution at the interface,^45^ which comprises both electrolyte and contact resistances.^45^ This finding highlights that the electrolyte dynamics are essential for achieving the quantum coherence value of *h*/2*e* ^2^ ≈ 12.9 kΩ at lower frequencies and room temperature.^45^ Furthermore, in the dynamic picture illustrated in Figure 1(c), the ‘drift’ velocity of this electrodynamics process can be estimated as ν_μ_ *L* ≈ 3 × 10^–8^ m s^–1^. This estimate confirms[Fn fn1] that ionic dynamics control the process, as they intertwine with the quantum transport of electrons.

### 3.2 Geobacter’s Respiration

The QR theory not only quantifies the ET rate of electrochemical reactions but also allows us to explain the long-range electron transport in respiration chains,^3^ as exemplified in Figure 5 that compares the interfacial ET between redox switch molecular films (<2 nm) and a (Geobacter) biological film (≈ μm), the latter serving as a model for studying respiration chains. Typically, this biological film serves as an example of long-range, coherent electron transport.^3^ Both situations follow *C*_q_ = *Ne* ^2^/(*k*_B_ *T*)*f*(1 – *f*), although, at a first look into the DOS shown in Figure 5(b), there are significant differences in the shape of that of Figure 5(a).^3^ This is because the occupancy of the biofilm defined by *f* in *C*_q_ follows a Boltzmann *f* = exp­(*eV*/*k*_B_ *T*) instead of Fermionic *f* = (1 + exp­(−*eV*/*k*_B_ *T*))^−1^ distribution.^3^

Accordingly, the only differences between the two settings are thermodynamics; the long-range thickness of the biofilm, where electrons are not confined (as is the case with the 2 nm thickness of the molecular film), establishes a different occupancy, which impacts the electron motion along with a long-range >50 μm distance.^3^ The different thermodynamics imply an occupancy in the biofilm that follows *C*_q_ = *C*_m_ exp­(*eV*/*k*_B_ *T*), where *C*_m_ = *e* ^2^ *N*/*k*_B_ *T* is the maximum capacitive occupancy of the biofilm, a limit which is shown as a straight green line in Figure 5(b).^3^

The analysis demonstrates that the respiration chain follows QR theory premises (a ν_μ_ = 1/τ ≈ 4 Hz was obtained for the respiration of the Geobacter) where the meaning of *C*_q_ is crucial for our understanding of the respiration mechanism,^3^ which eq 2, for instance, does not allow.

### 3.3 Low-Energy Electronic Spectroscopy: A Novel *In Situ* Characterization Method

The applications of QR theory extend beyond the study of ET reactions. As illustrated in Figures 6 and 7, QR theory enables us to investigate quantum coherent phenomena, even in the absence of ET reactions. This serves as the basis for understanding quantum (nanoscale) electronics operating within an electrolyte environment, demonstrating how quantum mechanics can function coherently at room temperature. These principles permit explanations for the quantum electrodynamics of graphene^60^ and the quantum mechanical basis of the supercapacitance phenomenon^28^ within graphene-based materials, where supercapacitance is predominant despite the absence of redox reactions. The electronic structure of low-dimensional scale materials assembled on electrodes can be accessed by measuring *E*_μ_ (the spectroscopic equivalent of *hν*_μ_) states at the interface.^51,52^ This spectroscopic principle has been demonstrated for quantum dots (QD) of (cadmium telluride) CdTe) [see Figure 6][Bibr ref51] and graphene [see Figure 7],^52^ where a ‘photon-like’ low-energy frequency perturbs *E*_μ_ states using the electrode as a probe, as illustrated in Figure 1(c). Notably, these spectroscopic principles operate because *C*_μ_ ∝ *C*_q_, which is proportional to the accessible DOS of different quantum moieties (QD or graphene).

**Figure 6:**
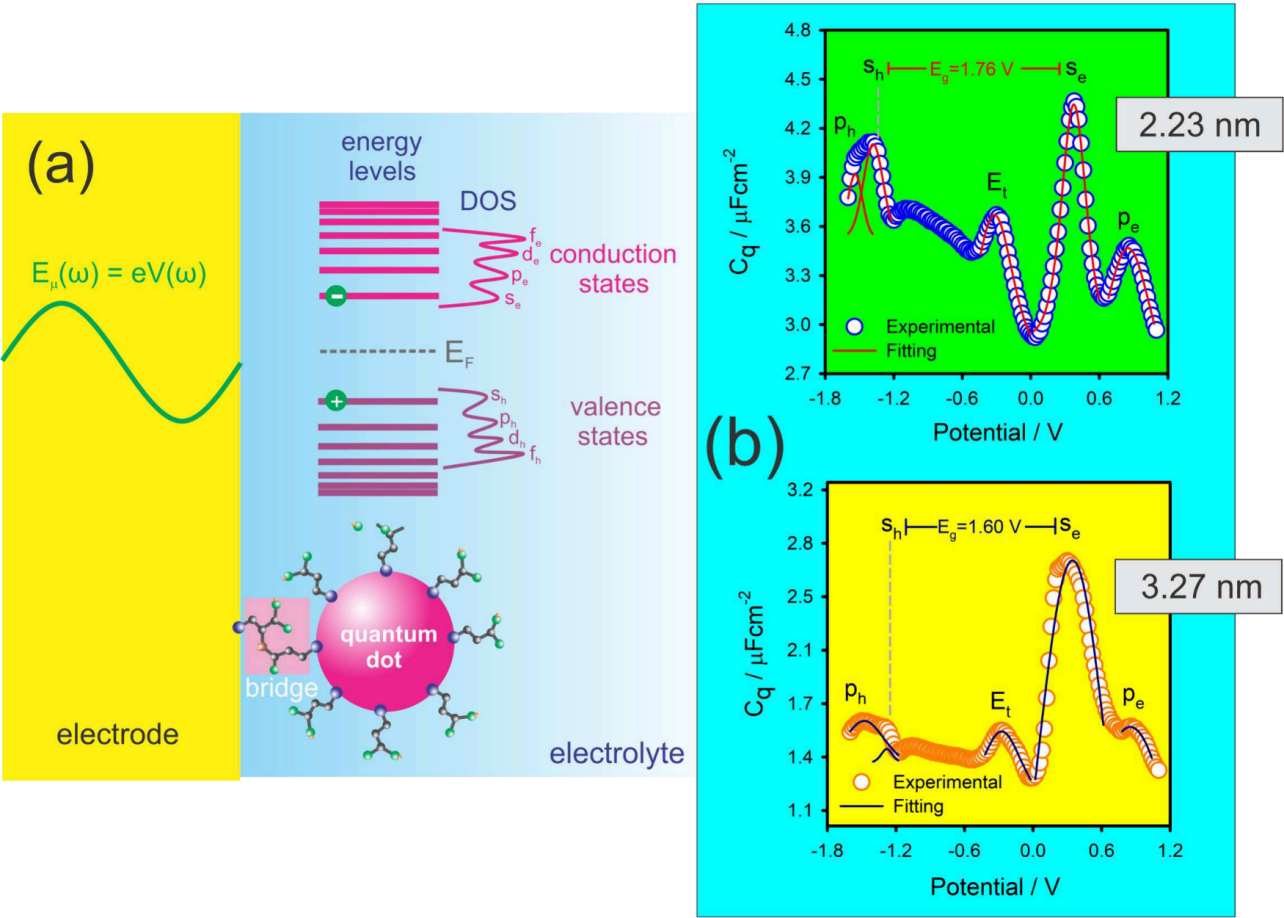
(a) Schematic representation of an electrode perturbation of an *E*_μ_ state comprising a CdTe QD. For QDs smaller than a certain limit (e.g., 5 nm), measurable electronic structure arises from the separation between energy levels, yielding discrete energy states that at a certain size resemble those observed in molecules (denoted here as conduction and valence states). (b) Electronic structure of two CdTe QDs of 2.23 and 3.27 nm, determined by QR spectroscopy as described in this text. The DOS shapes and spectra are comparable to results from the STM method. Reproduced (adapted) from ref 51 with permission from Edgar F. Pinzón, Las C. Lopes, André F. V. Fonseca, Marco A. Schiavon, and Paulo R. Bueno. Copyright (2024) Royal Society of Chemistry.

**Figure 7:**
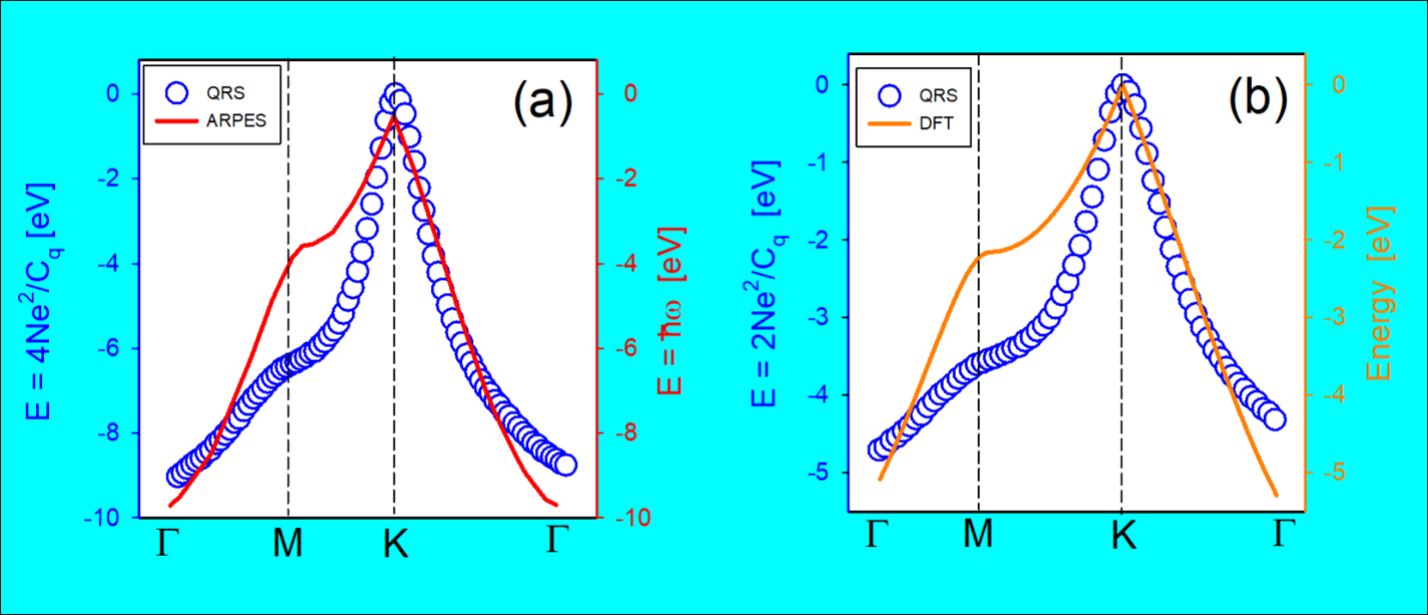
(a) Comparisons (including magnitude) of QR spectroscopy and ARPES methodologies for measurement of the electronic structure of graphene. The spectra are quite similar, and the minimal differences are associated with the QR spectroscopy being performed *in situ* at room temperature and in ambient electrolyte, whereas ARPES requires a low-temperature and vacuum environment. (b) The same as in (a), but the comparison is between the electronic structure obtained by QR spectroscopy and that calculated by the density functional theory computational methods. The calculated structure was for a vacuum and at zero temperature. Reproduced (adapted) from ref 52 with permission from Laís C. Lopes, Edgar F. Pinzón, Gabriela Dia-da-Silva, Gustavo T. Feliciano and Paulo R. Buen. Copyright (2024) Elsevier.

For instance, as shown in Figure 7(a), measurement of the electronic structure of graphene in an electrolytic, room temperature environment produced results comparable to those measured by ARPES, which requires ultrahigh vacuum and low temperatures. Similarly, measurement of the electronic structure of CdTe quantum dots (QD) under electrolytic conditions yielded results comparable to those obtained by scanning tunneling spectroscopy (STS), which also requires ultrahigh vacuum and temperature control.

Furthermore, the operational QR spectroscopic method relies only on the use of inexpensive electronic hand-held equipment and room-temperature electrolyte as a measurement medium, which can boost the design of miniaturized nanoscale electronics and electrochemical interfaces for a multitude of quantum physical and chemical electroanalyses, from medical diagnostics^61^ to drug discovery.^62^

### 3.4 Quantum Supercapacitance: The Graphene Oxide Case Study

Conventional interpretations of charge storage in supercapacitors often rely purely on electrostatic electric double-layer capacitance (EDLC). We challenge this view by demonstrating that the supercapacitance in Reduced Graphene Oxide (rGO) structures is fundamentally governed by QR theory premises in which *C*_e_ ≈ *C*_q_ are intertwined, providing a necessary link between ET kinetics and charge transport in nanosystems,^28^ which serves as a compelling application of QR theory premises to nanoscience and functional materials interfaces.

The observed increase in capacitance (∼173%) upon the electrochemical reduction of GO to rGO cannot be fully explained by an increase in electroactive surface area alone, indicating that considerations beyond purely geometric (EDLC) factors are required. Within the QR framework, the observed pseudocapacitance corresponds to the charging of the DOS, defined by *C*_q_/*e* ^2^, and is therefore proportional to *C*_q_. This is attributed to structural and electronic defects—such as vacancies and dangling bonds—created during the reduction of GO to rGO. According to QR theory, the total electrochemical capacitance *C*_μ_ results from the combined effects of the classical *C*_e_ and quantum *C*_q_ contributions, such that *e* ^2^/*C*_e_ ∼ *e* ^2^/*C*_q_. Capacitance is thus dominated by the occupation of *C*_q_ states, which is proportional to the accessible DOS.^28^

The ultimate proof of the quantum mechanical origin of rGO’s supercapacitance is found in the measurement of the charging resistance. The kinetics of charge storage is governed by the characteristic RC relaxation time, τ = *R*_q_ *C*_μ_, where *R*_q_ is the dynamic resistance that limits the rate ν_μ_ = 1/τ. Figure 8(b) displays two distinct quantum-resistive-capacitive *R*_q_ *C*_q_ contributions, each associated with a separate charging mode for two types of defects.

**Figure 8:**
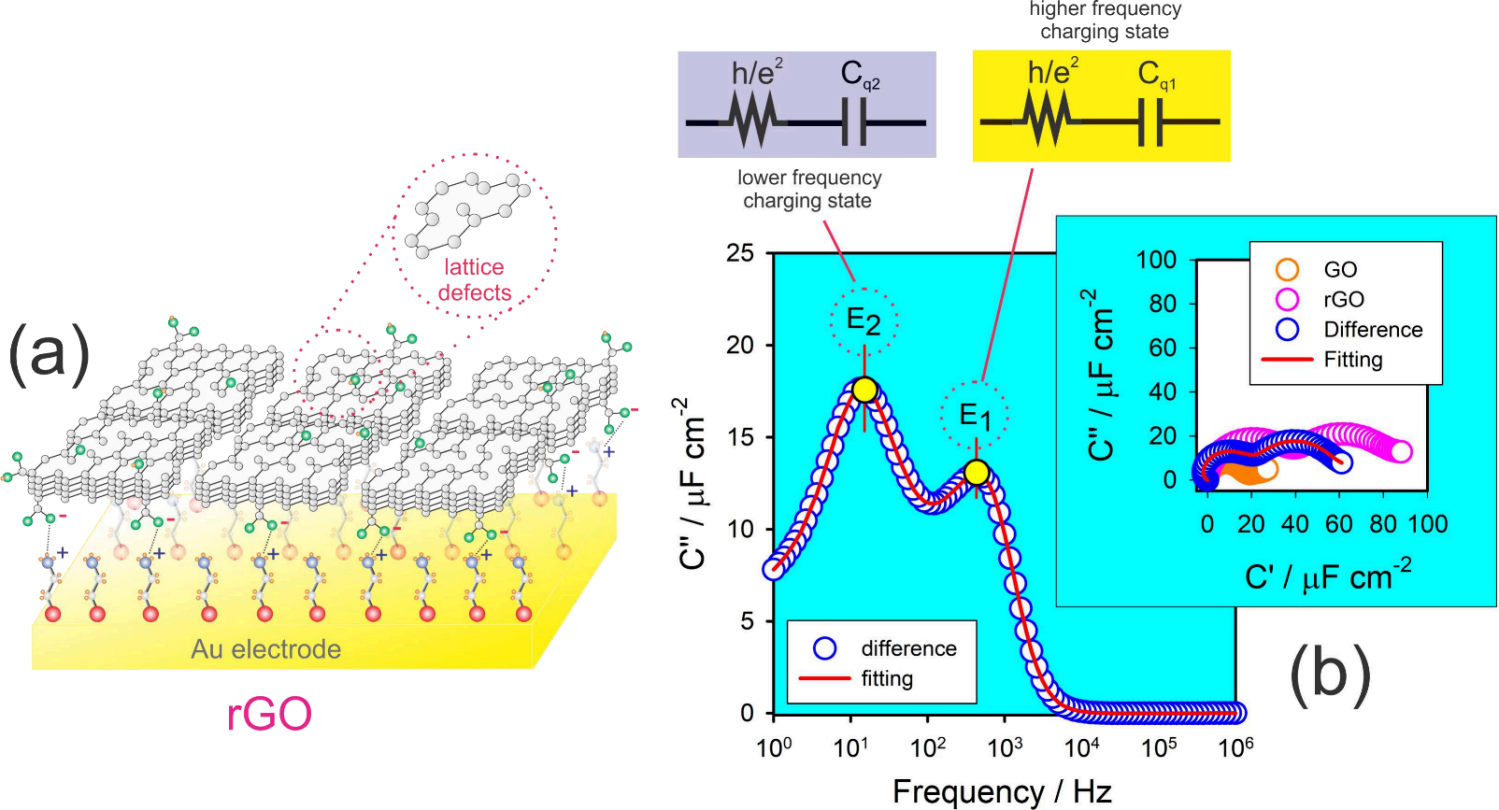
(a) Illustrates the rGO architecture immobilized on a gold electrode by a cysteamine self-assembled monolayer. Two charging quantum state regimes were investigated using the QR spectroscopic technique, as outlined in ref 28. (b) The spectroscopic characterization employs a complex admittance, G*­(ω). In the Bode plot, maxima of *C*″ versus frequency correspond to *E*_1_ = *e* ^2^/*hC*_q_1__ (modeled with an equivalent *R*_q_ *C*_q_1__ circuit) and *E*_2_ = *e* ^2^/*hC*_q_2__ (modeled as the *R*_q_ *C*_q_2__ circuit). Each quantum state is accessed from the electrode in a quantum-coherent (*h*/*e* ^2^) transport regime. *E*_2_ represents the dominant contribution to the supercapacitance response. The inset in (b) shows the Nyquist-type capacitive profile, depicting the rGO signature after subtraction of the GO background (purple opened circles), as specified in ref 28. Reproduced (adapted) from ref 28 with permission from Thamyres F. M. Moreira, Edgar F. Pinzón, Adriano dos Santos, Las C. Lopes, and Paulo R. Bueno. Copyright (2025) Elsevier.

Quantitative analysis of the complex capacitance spectrum using both traditional equivalent circuit fitting and spectroscopic subtraction [Figure 8(a)] revealed the existence of two chargeable electronic states in the rGO structure, *E*_1_ (higher frequency) and *E*_2_ (lower frequency).^28^

For the lower-frequency RC relaxation (*E*_2_), which represents the dominant pseudocapacitive contribution (Figure 8), the calculation of the characteristic quantum resistance *R*_q_ = τ/*C*_q_ yielded 25.6 kΩ. This value is within 1*%* error of the fundamental von Klitzing constant (*R*_K_ = *h*/*e* ^2^ ≈ 25.8 kΩ).^28^ This is a definitive, measurable signature that the dynamics governing charge injection and storage in rGO-based supercapacitors follows QR dynamics principles (*E* = *hν* ∝ *e* ^2^/*hC*_q_), directly challenging the classical, geometry-based view. The measurable parameters *R*_q_ and *C*_q_ thus provide a unified framework for characterizing both the kinetics (rate of charging) and the electronic structure (*C*_q_ ∝ DOS) of these high-performance nanosystems.^28^

## 4 Final Remarks and Conclusions

A theoretical principle, termed QR, that unifies ET rate (electrochemistry) and quantum transport (nanoscale electronics) phenomena operating in an electrolyte environment was revised. Namely, the QR viewpoint of these phenomena demonstrates how ET processes are driven by electrodynamics that obey quantum principles despite the damping of the electrolyte environment (classical dynamics). The electrolyte’s dynamics control the rate and ‘drift’ velocity of the process but permit quantum coherence (at ambient temperatures) to be maintained for the transport of electrons, which encompasses semiclassical ET transfer as a particular setting of the QR theoretical framework. *De facto*, it is the electrolyte dynamics that sets out the conditions for coherent transport to operate.

According to eq 4, coherent transport *R*_q_ = *g*_s_ *e* ^2^/*h* can be established between the quantum capacitance *C*_q_ and the electrode’s states. This occurs through an external temporal perturbation of the electrode provided that the electrode is modified with *E*_μ_ = *hν*_μ_ ∝ *e* ^2^/*hC*_q_ moieties, which are contained in low-dimensional structures and embedded in an electrolyte environment. The dynamics of the electrolyte is important because *E*_μ_ = *g*_e_(*e* ^2^/*C*_q_) = *g*_e_ *e* ^2^/*C*_e_, where the quantum and electrolyte states are superposed. This superposition maintains electroneutrality and electrochemical equilibrium. One can say this constitutes a particular isoscopic state, where classical and quantum mechanics meet. Therefore, the electrolyte environment is crucial for studying quantum electronics and designing quantum circuits that utilize quantum moieties, such as molecular switches.

The reorganization energy λ_0_, as defined in semiclassical electron transfer theory, must be reconsidered in the context of coherent quantum mechanics and electron transport between quantum states in electrolytes. Traditional definitions focus on ET rate constants in electrochemical reactions and do not incorporate electrolyte dynamics beyond the electron transfer phenomenon. However, some systems, such as those involving graphene and quantum dots discussed in section 3.3, lack ET reactions altogether. In these cases, coherent quantum electronics in electrolytes drive phenomena like push–pull junction electronics^63^ and supercapacitance^28^ in reduced and oxidized graphene. Experimental studies also reveal phenomena such as relaxation resistance *R*_q_ = *h*/*g*_s_ *e* ^2^ ≈ 12.9 kΩ (associated with *C*_q_), where ET is absent and the usual interpretation of λ_0_ does not apply. The QR framework replaces λ_0_ with measurable quantum circuit parameters (*R*_q_ and *C*_q_) intrinsic to the material’s electronic structure, providing a unified kinetic and transport view for electrochemical materials science. Therefore, further research is needed on the impact of electrolyte dynamics on quantum coherent transport to develop new theoretical frameworks that redefine λ_0_ beyond conventional ET chemical kinetics.

## Supplementary Material



## Footnotes

a The ‘drift’ velocity of ionic carriers is typically within millimeters per second or even millimeters per minute in solutions and electrolytes.

## Notes

Introduction to the Unification Framework; Foundational Concepts and Electrochemical Generalization; Recovery of the Semi-Classical Limit (Marcus ET). The Unifying Condition: Equivalence of λ_0_ and *E*_e_. References (PDF)
