# Supplementary material for: Quantum Rate Dynamics for Coherent Electron Transport at Material/Electrolyte Interfaces
Source: ACS Appl Mater Interfaces. 2026 Feb 13;18(7):10669–80. doi: 10.1021/acsami.5c25018 (PMC12954668; doi:10.1021/acsami.5c25018)
Supplement: Supplementary file 1 [file am5c25018_si_001.pdf]

SUPPORTING INFORMATION
Quantum Rate Dynamics for Coherent Electron Transport at Material/Electrolyte Interfaces

Paulo Roberto Bueno*

Department of Physics and Mathematics, Institute of Chemistry, São Paulo State University, Araraquara, 14800-060, São Paulo, Brazil

E-mail: paulo-roberto.bueno@unesp.br

1 Introduction to the Unification Framework

The purpose of this Supporting Information (SI) is to present the detailed mathematical and physical foundations that underlie the main text, specifically focusing on the derivation of the generalized quantum rate ν_μ and its statistical-mechanical properties. This explains the basis of **Quantum Rate (QR) theory**,[1,2] which unifies two perspectives: the **coherent electron transport** framework (from a physicist's viewpoint) and the **kinetic electron transfer** (ET) rate constant (from a chemist's viewpoint) in electrolytic environments. This unification is achieved by defining a generalized quantum rate, ν_μ, in terms of measurable quantum circuit parameters, namely the quantum resistance, R_q, and the electrochemical capacitance, C_μ.

2 Foundational Concepts and Electrochemical Generalization

2.1 Quantum Conductance and Resistance

The core of coherent electron transport is described by the **Landauer formula**, which defines the quantum conductance G based on transmission probabilities T_n through quantum channels:

$$G = G_0 \sum_{n}^{N} T_n \tag{1}$$

where $G_0 = g_s e^2/h$ is the conductance quantum. The total system quantum resistance R_q is the inverse of conductance: $R_q = 1/G$. In the context of the QR theory applied to molecular junctions, the experimentally measured total resistance, R_{total} (which includes dissipation from the electrolyte and contacts, $R_{total} = R_c + R_s$), is found to be precisely constrained to the fundamental quantum resistance value: $R_{total} = R_q = h/g_s e^2$ (approximately 12.9 kΩ for $g_s = 2$). This quantization of the total hybrid resistance is the experimental signature that the charge transfer process is fundamentally quantum-limited, despite the presence of classical resistance components.

2.2 Electrochemical Capacitance in the Electrolyte Environment

The total capacitance C_μ in an electrochemical system is the equivalent capacitance resulting from the series combination of the classical electrolyte capacitance C_e and the quantum capacitance C_q (related to the material's Density of States, DOS):

$$\frac{1}{C_\mu} = \frac{1}{C_e} + \frac{1}{C_q}. \tag{2}$$

This series relationship arises because charge accumulation in the electronic states (C_q)

and the necessary counter-charge accumulation in the ionic layer (C_e) must occur sequentially and simultaneously to respond to potential changes, limiting the total charge storage.

The electrochemical energy E_μ corresponding to this state is $E_\mu = e^2/C_\mu$.

2.3 The Generalized Quantum Rate ν_μ

The generalized quantum rate ν_μ is defined as the inverse of the characteristic electrochemical relaxation time τ: $\tau = R_q C_\mu$.

$$\nu_\mu = \frac{1}{\tau} = \frac{1}{R_q C_\mu} = \frac{G}{C_\mu}. \tag{3}$$

This definition applies fundamentally to the rate of a single electronic charge (e) traversing the junction, scaled by the total number of accessible channels. Substituting G from Landauer's formula (Eq. 1) and $1/C_\mu$ from the series combination (Eq. 2) leads to:

$$\nu_\mu = G_0 \left(\sum_n^N T_n \right) \left(\frac{1}{C_e} + \frac{1}{C_q} \right). \tag{4}$$

Recognizing $G_0 = g_s e^2/h$ and $1/C_\mu = E_\mu/e^2$, this simplifies to the direct relation between the rate and the electrochemical energy:

$$\nu_\mu = \frac{g_s}{h} \left(\sum_n^N T_n \right) E_\mu. \tag{5}$$

2.4 Coherent Transport Current and Conductance from Chemical Potential Difference

Figure 2 (of the main text) provides a description of the current i driven by the electrochemical potential difference $\Delta\mu = \mu_D - \mu_A$. This is the microscopic link between the applied potential, the electronic structure (DOS), and the resulting transport current. The current i is defined by:

$$i = -e\frac{c_*}{L}\Delta\mu\left(\frac{dn}{dE}\right), \tag{6}$$

where e is the elementary charge, L is the quantum channel length, $\Delta\mu = -eV$ is the electrochemical potential difference (driving voltage V), and (dn/dE) is the electronic Density-of-States (DOS), which is related to the Landauer conductance G (which includes coherence/transmission $\sum T_n$) by:

$$\frac{dn}{dE} = \frac{g_s L}{c_* h}\sum_n^N T_n.$$

Substituting this general DOS expression into the current equation (Eq. 6), we obtain:

$$i = -e\frac{c_*}{L}\Delta\mu\left(\frac{g_s L}{c_* h}\sum_n^N T_n\right),$$

which simplifies to:

$$i = -\frac{g_s e}{h}\left(\sum_n^N T_n\right)\Delta\mu$$

Finally, the conductance G is the ratio i/V and using the relation $\Delta\mu = -eV$, it leads to

$$G = \frac{i}{V} = \frac{-\frac{g_s e}{h}\left(\sum_n^N T_n\right)\Delta\mu}{-\Delta\mu/e} = \frac{g_s e^2}{h}\left(\sum_n^N T_n\right),$$

which ends to:

$$G = G_0\sum_n^N T_n. \tag{7}$$

This derivation confirms that the transport current i driven by the chemical potential difference $\Delta\mu$ is equivalent to the **Landauer quantum conductance** G. Since $R_q = 1/G$, the characteristic time $\tau = R_q C_\mu$ of the electrochemical system is fundamentally dictated by the quantum resistance, validating that the overall rate dynamics are constrained by the coherent quantum transport limit, as indicated in the main text.

3 Recovery of the Semi-Classical Limit (Marcus ET)

3.1 The Thermalized Quantum Rate $\nu_\mu(T)$

To account for thermal broadening of the quantum states, the rate of transfer between a donor (occupied state f) and an acceptor (available state $1-f$) is proportional to the product $f(1-f)$, where f is the Fermi-Dirac occupation number for a state with energy E at temperature T, $\beta = 1/k_BT$:

$$f(E) = \left[1 + \exp\left(\beta E\right)\right]^{-1}. \tag{8}$$

The full quantum-thermal rate $\nu_\mu(T)$ is written by normalizing the expression derived from the Density of States, and includes the degeneracy factors (g_s, for spin, and g_e, for C_e/C_q degeneracy):

$$\nu_\mu(T) = g_s g_e \frac{h}{k_BT} \cdot \left[\frac{G}{G_0}\frac{k_BT}{h}\right] \cdot \frac{1}{\beta k_BT} f(E)\left[1 - f(E)\right], \tag{9}$$

where G is the total quantum conductance (which is linked to the Landauer formula, $G = 1/R_q$, where R_q is the total quantized resistance) and $G_0 = g_s e^2/h$ is used to establish the correct dimensional normalization factor equivalent to the TST pre-factor. The dimensional factors $h/(k_BT)$ and $1/(\beta k_BT)$ cancel out (since $\beta k_BT = 1$). The resulting rate $\nu_\mu(T)$ is thus proportional to the quantum transmission $\sum T_n$ (contained in G) and the thermal broadening $f(1-f)$, directly connecting coherent transport to thermal kinetics.

3.2 The Boltzmann Approximation

The semi-classical limit, corresponding to highly activated processes (like the Marcus ET theory), assumes $\beta E \gg 1$. In this limit, the Fermi-Dirac distribution reduces to the Boltzmann distribution:

$$f(E) \approx \exp(-\beta E)$$
$$1 - f(E) \approx 1.$$

Substituting these approximations (and setting $g_s = g_e = 1$) into Eq. 9:

$$\nu_\mu(T) \approx \nu_{k,\text{eff}} \cdot f \approx \nu_{k,\text{eff}} \exp(-\beta E) . \tag{10}$$

Here, $\nu_{k,\text{eff}} = (G/G_0)(k_B T/h)$ represents the effective frequency term. The ratio $\kappa = G/G_0 = \sum T_n$ is the transmission coefficient, which, in the single-electron analysis limit, represents the probability of electron transfer. Thus, $\nu_{k,\text{eff}} = \kappa(k_B T/h)$, recovering the TST form where the frequency is scaled by the transmission κ. This result is the fundamental kinetic expression predicted by the semi-classical TST/Marcus ET theory, $k \propto \exp\left(-\beta E^{\ddagger}\right)$, confirming that the Marcus ET rate is a specific case (Boltzmann limit) of the generalized quantum rate description ν_μ.

3.3 Alternative Deduction: Rate Inverse to Thermal Broadening

An alternative mathematical deduction clarifying the inverse relationship between the quantum rate and the thermal broadening of the state can be conducted using the thermodynamic definition of quantum capacitance: $C_q = e^2(dn/dE)$. The thermal derivative of the occupation number (dn/dE) is related to the Fermi-Dirac product by:

$$\frac{dn}{dE} = \beta f(E) \left[1 - f(E)\right] . \tag{11}$$

By equating the two expressions for C_q and solving for the electronic energy $E = e^2/C_q$, it leads to

$$E = \frac{e^2}{C_q} = \frac{e^2}{e^2\beta f(1-f)} = \frac{1}{\beta f(1-f)} = \frac{k_B T}{f(1-f)}.$$

Substituting this energy expression into the fundamental Planck-Einstein relation $\nu = E/h$, and scaling by the overall degeneracy $g_s g_e$ and the TST pre-factor ν_k, we obtain:

$$\nu_\mu = g_s g_e \nu_k \left[f\left(1-f\right)\right]^{-1}. \tag{12}$$

To explicitly show the dependence on the measurable Landauer conductance (G) and its inverse (the total quantized resistance R_q), we define the transmission coefficient $\kappa = G/G_0$. We then substitute $\nu_k = \kappa(k_B T/h)$ into Eq. 12, yielding the analysis-friendly form:

$$\nu_\mu = g_s g_e \left[\frac{G}{G_0}\frac{k_B T}{h}\right]\left[f\left(1-f\right)\right]^{-1}. \tag{13}$$

The term $f(1-f)$ is the statistical variance of the Fermi-Dirac distribution, defined as $\sigma_f^2 = f(1-f)$, which quantifies the fluctuation in the occupation number of a quantum state due to thermal energy. The rate ν_μ is therefore inversely proportional to this variance, $\nu_\mu \propto (\sigma_f^2)^{-1}$. This explicitly shows that the kinetic rate is slowed by high thermal fluctuations (maximal broadening at $f \approx 0.5$). Importantly, the rate ν_μ is simultaneously proportional to the transmission coefficient $\kappa = \sum T_n$ (contained in G). Since the total experimental resistance $R_q = 1/G$ is found to be precisely quantized ($R_q = h/g_s e^2/\sum T_n$), the overall charge transfer remains dictated by the fundamental quantum constraint. This means that the reaction can be kinetically slow due to thermal broadening while still maintaining a high degree of **quantum coherence** in the transport mechanism. This distinguishes QR theory from purely classical approaches.

Both Eq. 12 and Eq. 13 expressions demonstrate that the quantum rate is inversely proportional to the thermal broadening term $f(1-f)$. The slowest rates occur when thermal broadening is maximal ($f \approx 0.5$), whereas the fastest rates occur when it is minimal, confirming the link between low-frequency kinetics and thermal distribution of quantum states.

4 The Unifying Condition: Equivalence of λ_0 and E_e

The optimal "activationless" ET rate, where the reaction proceeds fastest, is achieved under the condition $E^{\ddagger} = 0$.

4.1 Marcus Condition

In Marcus theory, $E^{\ddagger} = 0$ occurs when the standard free energy of reaction E^0 is perfectly balanced by the reorganization energy λ_0:

$$E^0 = -\lambda_0 \tag{14}$$

4.2 QR Theory Equivalent

In the QR theory, this same physical balance is achieved when the electronic energy E is balanced by the energy contribution from the electrolyte E_e:

$$E = -E_e \tag{15}$$

Since $E = e^2/C_q$ and $E_e = e^2/C_e$, the condition $E = -E_e$ implies the **physical equivalence** between the reorganization energy λ_0 (chemists' parameter) and the measurable electrolyte energy parameter E_e (physicists' parameter) under the optimal rate condition.

4.3 Final Unification Formula and Conclusion

The simplest formula demonstrating the unification between **electron transport** and **electron transfer rate** is derived directly from the definition of the quantum rate (Eq. 3), by setting $\nu_\mu = k$:

$$G = kC_\mu \tag{16}$$

This relation formally links the **coherent quantum conductance** G (Landauer formalism) to the **kinetic rate constant** k (Marcus formalism), mediated solely by the **electrochemical capacitance** C_μ, which encapsulates the combined electronic and electrolytic environment effects.

The QR theory thus replaces the phenomenological parameter, the **reorganization energy** (λ_0), with the pair of directly measurable quantum circuit parameters: the electronic **quantum capacitance** (C_q) and the **electrolyte capacitance** (C_e), which together determine the rate constant and the overall energy dynamics of the electrochemical system.

References

(1) Bueno, P. R. Quantum rate theory and electron-transfer dynamics: A theoretical and experimental approach for quantum electrochemistry. *Electrochimica Acta* **2023**, *466*.

(2) Bueno, P. R. *Nanoscale Electrochemistry of Molecular Contacts*; Springer, 2018.
